# The Social Context of “Do-It-Yourself” Brain Stimulation: Neurohackers, Biohackers, and Lifehackers

**DOI:** 10.3389/fnhum.2017.00224

**Published:** 2017-05-10

**Authors:** Anna Wexler

**Affiliations:** Department of Science, Technology and Society, Massachusetts Institute of TechnologyCambridge, MA, USA

**Keywords:** non-invasive brain stimulation, transcranial direct current stimulation (tDCS), consumer neurotechnology, do-it-yourself, DIY biology, quantified self, cognitive enhancement

## Abstract

The “do-it-yourself” (DIY) brain stimulation movement began in earnest in late 2011, when lay individuals began building stimulation devices and applying low levels of electricity to their heads for self-improvement purposes. To date, scholarship on the home use of brain stimulation has focused on characterizing the practices of users via quantitative and qualitative studies, and on analyzing related ethical and regulatory issues. In this perspective piece, however, I take the opposite approach: rather than viewing the home use of brain stimulation on its own, I argue that it must be understood within the context of other DIY and citizen science movements. Seen in this light, the home use of brain stimulation is only a small part of the “neurohacking” movement, which is comprised of individuals attempting to optimize their brains to achieve enhanced performance. Neurohacking itself is an offshoot of the “life hacking” (or “quantified self”) movement, in which individuals self-track minute aspects of their daily lives in order to enhance productivity or performance. Additionally, the home or DIY use of brain stimulation is in many ways parallel to the DIY Biology (or “biohacking”) movement, which seeks to democratize tools of scientific experimentation. Here, I describe the place of the home use of brain stimulation with regard to neurohackers, lifehackers, and biohackers, and suggest that a policy approach for the home use of brain stimulation should have an appreciation both of individual motivations as well as the broader social context of the movement itself.

## Introduction

The “do-it-yourself” (DIY) brain stimulation movement began in earnest in late 2011, when lay individuals began building stimulation devices and applying low levels of electricity to their heads for self-improvement purposes (Wexler, 2016b). Today, in lieu of constructing their own devices, most individuals purchase one of more than a dozen different direct-to-consumer apparatuses that are available to the general public. As some use the phrase “DIY brain stimulation” to refer to the actual construction of the device and others use it in reference to the unsupervised nature of stimulation itself, here I adopt the term “home users” to describe those who stimulate their own brains outside of medical and academic settings.

It has now been 5 years since home users began utilizing brain stimulation, and the anniversary marks a critical juncture at which to assess the movement's overall trajectory. Indeed, a number of points have become clear: first, while some predicted that the home use of brain stimulation would become widespread, the practice has yet to gain a foothold amongst the general public. Rather, it has remained a subculture, likely fueled in part by media coverage in major outlets like *Radiolab, Wired* and the *New Yorker*. Second, informal observations have suggested that the attrition rate among home users appears to be significant: many home users who I interviewed several years ago are no longer actively using stimulation, and there is a large amount of turnover on the Reddit.com forum dedicated to transcranial direct current stimulation (tDCS). Thus, it remains to be seen whether tDCS is something that users utilize fleetingly or whether it has true staying power. Third, while no significant adverse events have been reported among home users of brain stimulation on Internet forums, tingling, headache, dizziness, and skin redness are common side effects (Jwa, 2015), and there have been occasional mentions of skin burns^1^.

To date, scholarship on the home use of brain stimulation has focused on characterizing the practices of users via quantitative and qualitative studies (Jwa, 2015; Wexler, 2016b), and on analyzing related ethical (Hamilton et al., 2011; Cohen Kadosh et al., 2012; Cabrera et al., 2013) and regulatory issues (Fitz and Reiner, 2015; Maslen et al., 2015; Wexler, 2016a). In this perspective piece, however, I take the opposite approach: rather than viewing the home use of brain stimulation on its own, I argue that it must be understood within the context of other DIY and citizen science movements. Seen in this light, the home use of brain stimulation is only a small part of the “neurohacking” movement, which is comprised of individuals attempting to optimize their brains to achieve enhanced performance. Neurohacking itself is an offshoot of the “life hacking” (or “quantified self”) movement, in which individuals self-track minute aspects of their daily lives in order to enhance productivity or performance (Swan, 2013). Additionally, the home or DIY use of brain stimulation is in many ways parallel to the DIY Biology (or “biohacking”) movement, which seeks to democratize tools of scientific experimentation (Delfanti, 2013). Here, I describe the place of the home use of brain stimulation with regard to neurohackers, lifehackers, and biohackers, and suggest that it is only by viewing the movement within broader social and cultural contexts that ethical and regulatory questions can be appropriately understood and addressed.

## Brain enhancement in the digital age: neurohackers

Since the turn of the twenty-first century, neuroscience has had an increasing place in the public realm (O'Connor, 2013; Rose and Abi-Rached, 2013). Empirical studies have examined representations of neuroscience (and neuroimaging) in the media (Racine et al., 2005, 2006, 2010; O'Connor et al., 2012), as well as the alluring appeal of neuroscience explanations (Weisberg et al., 2008). In the early 2000s a flood of popular science books, such as *The Brain that Changes Itself*, helped catapult neuroscience into the self-help arena, and the neuroscience concept of “plasticity” was co-opted into a mantra for personal growth and spirituality. Rather than a passive organ that exists as an immutable part of our bodies, the brain has been increasingly conceptualized as something to be exercised, re-shaped, and maximized. Indeed, a study of brain-related articles published in six United Kingdom newspapers between 2000 and 2010 found that 43% characterized the brain as a “resource to be optimized” (O'Connor et al., 2012).

In an effort to capitalize on the brain optimization trend, a number of companies—such as Lumosity, PositScience, and CogniFit—began selling brain-training software games. The products emphasized the idea of brain fitness, and were marketed to those wanting to stave off age-related cognitive decline or even dementia. Though the generalizability of brain-training games to other domains has repeatedly been called into question (e.g., Au et al., 2016; Foroughi et al., 2016), and the Federal Trade Commission has recently taken action against several companies for misleading marketing claims (FTC, 2016a,b), the brain-training game market is still thriving, with $67 million in sales in North America in 2015 alone (Sparks, 2016).

Even without training, the brain has come to be viewed as something that can be enhanced—via drugs (both legal and illegal), dietary supplements, food, drinks, and even chewing gum. Websites and forums have sprung up dedicated to the world of “nootropics,” a term used to describe “smart drugs” and dietary supplements that (supposedly) improve one's intelligence or cognitive ability. Today, the Reddit.com forum on nootropics has 80,000 subscribers and averages several posts daily. More recently, Silicon Valley start-ups like Nootrobox (tagline: “nootropics for everyone”) have sought to bring brain-boosting supplements to the mainstream.

In parallel to the commercialization of “brain optimization” games and nootropics, a number of companies began developing pared-down versions of neuroscience tools, such as electroencephalography (EEG) devices, and marketing them directly to consumers^2^. Although some direct-to-consumer EEG products initially focused on mind-control applications, such as the ability to move a computer cursor using one's thoughts, today the majority are geared toward brain optimization and wellness. Reflecting the rise of what has become known as “neurotechnology,” a variety of industry groups, independent market research firms, and non-profit organizations now hold conferences and meetings related both to direct-to-consumer and clinical applications of neurotechnology^3^.

The rise of brain training, the increasing availability of brain-enhancing drugs and supplements, and the commercialization of neuroscience tools all set the foundation for the emergence of DIY and direct-to-consumer brain stimulation. Viewed myopically, the home use of brain stimulation can be perceived as individuals merely adapting scientific techniques for use on themselves. But considered more broadly, the movement is part-and-parcel of the “neurohacking” movement, and cannot be fully comprehended without understanding the overall drive for brain enhancement.

Yet compared to other “neurohacking” techniques, the lay use of brain stimulation represents only a very small piece of the puzzle: the Reddit forum on nootropics has 10 times the number of subscribers as its tDCS counterpart; and there are dozens, if not hundreds, of companies selling brain-boosting nootropics, compared to roughly a dozen direct-to-consumer tDCS companies (the majority of which are individuals building and selling devices from home in their spare time). Although there are no official estimates regarding the size of the consumer brain stimulation market, based on my interviews with manufacturers of brain stimulation devices, it seems that tens of thousands of devices have been sold in the last several years—though current users may only comprise a small proportion of that figure. By comparison, a single brain-training company, Lumosity, reported having 70 million subscribers in 2015 (Lumosity, 2015). Thus, as will be discussed below, when developing policies and regulations for the home use of brain stimulation, it may be prudent not to focus solely on stimulation but to develop frameworks that encompass direct-to-consumer enhancement products as a whole.

## Citizen science and the democratization of scientific tools: biohackers

When DIY brain stimulation first emerged, some suggested adopting an open-engagement approach akin to that taken to DIY Biology (Fitz and Reiner, 2015), which is a movement of makers and tinkerers doing biology in kitchens and garages (Ledford, 2010; Roosth, 2010; Wohlsen, 2012; Delfanti, 2013; Delgado, 2013; Meyer, 2013; Sanchez, 2014). The proposition has, to some extent, come to fruition: a recent open letter authored by four neuroscientists (and signed by 39 other researchers) directly addressed home users, and in measured tones, outlined the unknown risks of brain stimulation (Wurzman et al., 2016).

Indeed, the two movements share many similarities: like home users of brain stimulation, those who identify with DIY Biology are interested in democratizing the tools of science, and use an online forum (a Google group) as their main nexus of interaction. The DIY Biology forum discussions, like those on the Reddit.com tDCS forum, center on topics such as acquiring tools to carry out home experiments, sourcing inexpensive equipment, and constructing affordable versions of laboratory tools. Both movements rely heavily on scientific counterparts: home users look to scientific articles for guidance (and frequently link to them online) and DIY biologists rely on the infrastructure built up by synthetic biology (Roosth, 2010).

Members of both movements embody what has become known as the maker culture, which places a high value on tinkering, engineering, and creating things from scratch. There are ongoing debates on the DIY biology list about what is considered “real” DIY: Roosth (2010, p. 126–127) writes that once “an argument broke out about whether it was truly “DIY” to order gel starter kits and other readymade products from biological supply companies.” Similar debates took place in the DIY tDCS community after the release of the first direct-to-consumer devices: those who purchased ready-made devices were derided for not doing sufficient “homework” to understand electrical circuitry.

But a deeper excavation of the underlying goals of each community reveals fundamental differences. The primary goal of home users of brain stimulation, whether self-treating for depression or attempting to enhance their memory, is self-improvement. By contrast, the goal of DIY biology is more political in nature: DIY biologists talk about redistributing power and fundamentally revising the way that science is done (Delgado and Callen, 2016). A “biopunk manifesto” written by one DIY biologist asserts: “We the biopunks are dedicated to putting the tools of scientific investigation in the hands of anyone who wants them” (Patterson, 2010, as quoted in Delfanti, 2013, p. 126). According to Delfanti (2013, p. 115), “right now citizen biology is not a site of research and innovation but rather of political, artistic, and educational experimentation.”

While in many ways the home use of brain stimulation is an inherently political act—against the scientific community's tendency to restrict knowledge and devices to a privileged few—the movement is characterized by a culture of deference and respect toward scientific institutions, as it is the scientific knowledge that will ultimately help users achieve their self-improvement goals. But in DIY Biology, it is politics, not self-improvement, that takes center stage. As Roosth (2010, p. 112) writes: “biohackers do not pursue or promote science as a path to personal improvement or refinement, but as a pleasure and a kind of political speech.” Thus while DIY biologists and home users may appear rather similar at the surface level—in terms of what they talk about, how they talk about it, their reliance on science and their existence on the edges of it—their underlying goals are in fact quite different. Therefore, when considering policy approaches for the various DIY and citizen science movements, it is crucial to understand the goals and motivations of each, no matter how similar they may outwardly appear.

## Digital tracking and quantified self: lifehackers

Like home users of brain stimulation, those who identify with the “life hacking” or quantified self (QS) movement have the ultimate goal of enhancing themselves; they track various aspects of their lives in order to improve (or “hack”) them (Lupton, 2013; Swan, 2013; Selke, 2016). Lifehackers, more commonly known as self-trackers or QSers, place a high value on the collection and analysis of information; data is thought to illuminate knowledge of the self.

Self-trackers frequently engage in forms of self-experimentation. For example, they may hypothesize about which factors affect their cognition or mood (Roberts, 2004, 2010): does drinking coffee after 3 p.m. cause them to stay up late, and does eating Bleu cheese make them more alert? Self-trackers test such hypotheses, often plotting the resulting data on graphs and attempting to derive meaning from them. When it comes to data analysis, they struggle with the same issues as home users of brain stimulation: namely, the methodological limitations of a sample size of one. To date, self-trackers have not published aggregated data on their experiments in a peer-reviewed journal.

As a whole, self-trackers are a more coherent and organized group than home users of brain stimulation. In addition to annual international and regional “quantified self” conferences, there are over 200 groups in more than 30 different countries where individuals meet to share the results of their self-experimentation data, presenting what they did, how they did it, and what they learned (Nafus and Sherman, 2014; Barta and Neff, 2016). In contrast, home users of brain stimulation have never coalesced into an official group, and there has been no formal gathering of any kind (Wexler, 2016b).

Home users of brain stimulation share the same ethos as self-trackers: namely, they experiment on themselves, report their methods, and share their results, no matter how personal or private. However, home users focus narrowly on two forms of self-improvement—cognitive enhancement and/or self-treatment—using a single intervention, that of non-invasive brain neurostimulation. Self-trackers are interested more broadly in productivity, mood and performance. For them, an intervention is not always required—often insight can be gained merely by analyzing self-tracking data. There is likely overlap between the groups, and at least some self-trackers have also tried brain stimulation: Dave Asprey, a prominent figure in the lifehacking movement, included a direct-to-consumer brain stimulation device in a recent shipment to subscribers of his quarterly productivity package.

Like neurohacking, self-tracking has become commercialized and commodified: whereas users once entered data manually into Excel spreadsheets, today there are hundreds, if not thousands, of mobile apps and wearable sensors that facilitate the tracking of sleeping patterns, fitness levels, eating habits, productivity, and mood. Activity monitors like the FitBit and Jawbone have become commonplace, and new iPhones come pre-loaded with Apple's suite of health-monitoring applications. Though some of these technologies are geared to those managing illnesses, they are marketed equally as “wellness” products to healthy individuals. As Schüll (2016, p. 3) puts it, there is now a culture of “data for life” where our own wellness “depends on the continuous collection, analysis and management of personal data through digital sensor technologies.”

## Discussion

Viewed broadly, the home use of brain stimulation sits at the nexus of maker and DIY cultures, citizen science movements, and self-experimentation and self-tracking initiatives. Like “biohackers,” home users source inexpensive versions of restricted laboratory tools for use at home; like “life hackers,” they are primarily interested in self-improvement. Though the home use of non-invasive brain stimulation has received a significant amount of attention in both media outlets and scholarly journals, it remains a very small part of the overall “neurohacking” movement, wherein individuals aim to optimize their brain function.

Although here I have discussed neurohackers, biohackers, and lifehackers separately, it is important to note that there is fluidity amongst the terms: for example, some use “life hackers” and “biohackers” interchangeably to refer to DIY Biology, whereas others use “biohackers” to refer only to those who physically modify their bodies for self-improvement purposes. Furthermore, those who use commercial wellness or self-tracking products—like the Muse headset or a FitBit—may not self-identify with any kind of “hacking” movement.

How can situating the home use of non-invasive brain stimulation within broader contexts help inform regulatory and ethical issues? First, it can help maintain an awareness of the larger policy question at hand related to “neurohacking,” which is how to regulate the entire ecosystem of products (e.g., brain stimulation devices, EEG recording devices, brain-training games) marketed directly to consumers to enhance their brain function. For example, should “cognitive enhancement” products be considered similar to medical devices and therefore regulated by Food and Drug Administration (FDA)? Or should they be conceptualized closer to “wellness” or “fitness” products, and therefore subject to a less stringent degree of oversight from authorities such as the Consumer Product Safety Commission (CPSC) or the Federal Trade Commission (FTC)? As I have argued previously (Wexler, 2016a), enforcement clarity is needed to establish which agencies will exercise primary authority over these products.

In addition, explicitly identifying movements and locating the connections between them can help draw together scholars studying parallel phenomenon from disparate disciplines. This paper has drawn on literature from sociology, bioethics, STS (science, technology, and society), and neuroscience; however, DIY technologies and citizen movements are also being examined from legal, medical, economic, and securities studies perspectives, among others (see, e.g., Wang and Kaye, 2011; Doherty, 2012; Bolton and Thomas, 2014; Bryans, 2015; Walther, 2015; Snow, 2015; Lee et al., 2016; Awori and Lee, 2017). Across disciplines, scholars use different frameworks and language to describe similar phenomenon—for example, conceiving of DIY techniques as “radical leveling technologies” (Snow, 2015) or studying how they “democratize innovation” (von Hippel, 2009). In the case of neurohackers, biohackers, and lifehackers, it may be fruitful to bridge traditional disciplinary divides and share insights to better understand how these movements work.

While it is important to keep in mind the overall similarities *across* movements, this perspective piece has also focused on describing goals and motivations *within* movements. Indeed, a sociological and cultural awareness of the individual movements themselves is essential for the development of sound policy, as it can help predict the potential outcome of regulation. For example, a heavy-handed regulatory approach to non-invasive brain stimulation might have the effect of drawing more attention to a movement that may not be particularly large to begin with (this phenomenon has been described as the “Streisand effect,” see Jansen and Martin, 2015). Furthermore, knowledge of users' motivations for using non-invasive brain stimulation might be useful in terms of predicting whether or not users might go “underground” in response to regulation; for example, those using brain stimulation to treat depression may be more likely to do so than those using the technology for enhancement. Economists have described a variety of other unwanted effects that can occur when regulations are set forth with a lack of cultural and sociological awareness, see, e.g., “compliance without effect” as described in Bryans (2015, p. 909) and “motivation crowding” (see, e.g., Frey and Jegen, 1999). Ultimately, a policy approach for the home use of brain stimulation should have an appreciation both of individual motivations as well as broader social contexts.

The present discussion has also highlighted the trend toward the commercialization of DIY tools and techniques for the optimization of performance. Although scholarship to-date has discussed many of the ethical issues that arise from the home use of brain stimulation, there are additional complications that may arise when such tools are commercialized and sold to others. For example, even beyond the requirements of the law, do companies have an ethical responsibility to guide consumers toward responsible and safe uses of neurostimulation devices, or to caution against their use in vulnerable populations? Given that the number of companies manufacturing direct-to-consumer enhancement technologies is likely to only increase, it may be fruitful for neuroethicists to collaborate with business ethicists, who study the ethical responsibilities of corporations outside of legal ones.

The present perspective piece has attempted to situate the home use of brain stimulation in its broader social milieu, providing context for the rise of the phenomenon and describing its parallels to concurrent cultural movements. Sociologists have long emphasized that social phenomena do not exist in isolation; rather they sit in relation to other phenomena, and cultural memes circulate across domains. As I have shown here, the home use of brain stimulation did not emerge from the ether, but arose from the confluence of DIY and citizen science movements, neuroenhancement initiatives, and a culture of self-tracking.

## Author contributions

AW conceived of and wrote this article.

### Conflict of interest statement

The author declares that the research was conducted in the absence of any commercial or financial relationships that could be construed as a potential conflict of interest.

## Footnotes

^1^See, e.g., https://www.reddit.com/r/tDCS/comments/2kw20k/serious_burn_using_external_electrodes_with_focus/; https://www.reddit.com/r/tDCS/comments/2c1g63/serious_burns_from_focus/; and https://www.reddit.com/r/tDCS/comments/55r1hn/new_to_tdcs_is_the_red_circle_burn_normal/.

^2^See, e.g., Emotiv Insight (https://www.emotiv.com/insight/); Muse (http://www.choosemuse.com/); and Melon (https://web.archive.org/web/20150318002158/http://www.thinkmelon.com/).

^3^See, e.g., Neurotechnology Industry Organization (https://www.neurotechindustry.org/); NeuroInsights (https://www.neuroinsights.com/), NeuroTechX (http://neurotechx.com/). See also SharpBrains (http://sharpbrains.com/).
